# What Are the Important Health and Well‐Being Outcome Dimensions for Parent Carers of Disabled Children? A Qualitative Study

**DOI:** 10.1111/hex.70358

**Published:** 2025-07-29

**Authors:** Caomhán McGlinchey, Phillip Harniess, Aleksandra J. Borek, Alice Garrood, Annabel McDonald, Fleur Boyle, Stuart Logan, Christopher Morris

**Affiliations:** ^1^ Peninsula Childhood Disability Research Unit (PenCRU) University of Exeter Medical School University of Exeter Exeter UK; ^2^ NIHR Applied Research Collaboration South West Peninsula (PenARC) University of Exeter Medical School University of Exeter Exeter UK; ^3^ Nuffield Department of Primary Care Health Sciences, Medical Sciences Division University of Oxford Oxford UK; ^4^ Institute of Psychology SWPS University Wroclaw Poland

**Keywords:** health, parent carers, patient‐reported outcome measures, PROM, qualitative, well‐being

## Abstract

**Background:**

Patient‐reported outcome measures (PROMs) are used to evaluate the effectiveness of interventions, particularly for subjective health states such as well‐being. A parent carer is an adult primary caregiver for a child with a disability. Parent carers are at risk of poorer mental and physical health; targeted health promotion interventions are being developed, requiring evaluation. This study aimed to identify the important health and well‐being outcomes for parent carers for evaluating parent carer‐focused interventions.

**Methods:**

We recruited parents of children with special educational needs and disabilities living in England. Participants took part in individual, semi‐structured interviews via video in two parts. First, participants were asked open‐ended questions about health and well‐being; second, there was an elicitation exercise, in which the interviewees were asked to consider the aspects of health assessed by the Warwick‐Edinburgh Mental Wellbeing Scale (WEMWBS). Verbatim transcripts were analysed in two stages. Initially, data were analysed inductively, taking a thematic analysis approach. Subsequently, the data were analysed deductively with reference to a comprehensive framework of well‐being composed of 6 domains and 196 dimensions of well‐being.

**Results:**

Thirty parent carers participated. We found support for diverse dimensions across all six well‐being domains. Eighteen dimensions were perceived to be of greater importance for parent carers: ‘stress reaction’, ‘anxiety/depression’, ‘acceptance’, ‘autonomy’, ‘self‐esteem’, ‘cognition’, ‘achievement’, ‘interests/hobbies’, ‘learning’, ‘need for relatedness’, ‘rest’, ‘sleep’, ‘physical exercise’, ‘life purpose and satisfaction’, ‘community wellbeing’, ‘status’, ‘financial situation’ and ‘future security’. WEMWBS items focus on positive aspects of mental health. Not measuring reduction in ‘negative’ well‐being dimensions risks missing important changes.

**Conclusions:**

All six domains of health and well‐being were relevant to parent carers. However, some dimensions were more significant for the well‐being of parent carers, and these dimensions should be captured when evaluating their health and well‐being outcomes.

**Patient or Public Contribution:**

Parent carers were involved in all aspects of the research, including research aims, recruitment and sampling, data collection and analysis, and dissemination.

## 1 Introduction

A ‘parent carer’ is an adult primary caregiver for a child with a disability with parental responsibility. There is substantial evidence from several countries that parent carers of children with chronic or life‐limiting conditions are at risk of poorer physical and mental health than parents of typically developing children [1, 2, 3]. To address this health need, a growing number of parent carer‐focused interventions have been designed that aim to improve their health and well‐being. Examples include Early Positive Approaches to Support [4], HOPE Programme [5], Healthy Mothers, Healthy Families [6], and Healthy Parent Carers (HPC) [7, 8].

Patient‐reported outcome measures (PROMs) are commonly used to measure the effectiveness of health and well‐being interventions in evaluative research. Our recent scoping review identified 133 different standardised PROMs used to evaluate 145 different parent carer‐focused interventions [9]. The most used measures were the Family Empowerment Scale, Parenting Stress Index, Parenting Sense of Competence Scale, Warwick‐Edinburgh Mental Wellbeing Scale (WEMWBS), Depression Anxiety and Stress Scale, General Health Questionnaire, and Pediatric Quality of Life Family Impact Module. Evidently, each of these and the other measures identified evaluates different concepts defined by the developers of the measures when establishing content and construct validity. These PROMs also represent a mix of generic and parent/family‐specific measures, reflect positive and negative aspects of health, and focus either on individual and/or family outcomes.

When selecting outcome measures for evaluative research, useful resources include online catalogues of PROMs such as PROQOLID (eprovide.mapi‐trust.org/about/about‐proqolid), and tools for appraising the measurement properties of PROMs, such as COSMIN resources (COnsensus‐based Standards for the selection of health Measurement Instruments, www.cosmin.nl) [10]. It has been highlighted that researchers have not always measured the outcomes that are most important to participants, leading to avoidable waste in research and misleading interpretations about the effectiveness of interventions [11]. This highlights the importance of the criterion of ‘appropriateness’ when selecting PROMs for trials, alongside reliability, validity, responsiveness, precision, interpretability, acceptability and feasibility [12].

Appropriateness requires consideration of the match of a PROM to the specific purpose and questions of the evaluative research [12]. Therefore, when selecting outcome measures to assess change in health and well‐being, as opposed to parent empowerment or competence in our example, the questionnaire items and response options must reflect matters of importance to the people in the study to ensure they will be sensitive to important change [12]. Approaches for considering appropriateness include those that are theoretical by linking to the logic model for short‐ and long‐term targets and consultation with people like the prospective participants, as patient and public involvement (PPI) in research.

HPC is a peer‐led, group‐based health promotion intervention for parent carers of disabled children [7]. Our feasibility randomised controlled trial of HPC delivered in person before the Covid‐19 pandemic used the WEMWBS as the putative primary outcome [13, 14, 15]. The feasibility trial demonstrated that we could recruit and train facilitators and that they could deliver the programme with fidelity and high ( > 80%) participant retention [16]. Progression criteria in our feasibility trial were met, making it reasonable to proceed to a definitive trial that can evaluate effectiveness and cost‐effectiveness [16]. Nevertheless, we were concerned that the positive and substantial benefits of HPC reported in our process evaluation were not reflected in changes to the distribution of responses to individual WEMWBS items pre‐ and post‐intervention [16, 17]. Investigating these concerns is important not only for HPC but also valuable for evaluations of other parent carer‐focused interventions and also demonstrates methodological approaches to due diligence when selecting PROMs in any context.

Previously, using an inductive thematic analysis approach, we described how factors contributing to poor health outcomes among parent carers can result from common stressors experienced by the parent carer population [18]. In this study, we sought to classify the aspects of health and well‐being that are most salient and important to parent carers with reference to a comprehensive framework of well‐being dimensions [19]. We also considered the appropriateness of WEMWBS as a measure of well‐being for parent carers.

## 2 Methods

### 2.1 PPI

Our Family Involvement Coordinator (A.Mc.D.) co‐facilitated a study‐specific PPI group. A comprehensive description of our PPI involvement is provided in Table 1, following the Guidance for Reporting Involvement of Patients and the Public (GRIPP‐2) [20].

**Table 1:** Public and patient involvement (PPI) in this study.

Topic	Item
Aims	To ensure that the study remained focused on the parent carer perspective in addressing its main aim. To identify the health and well‐being outcomes most salient to parent carers—supporting researchers interested in measuring health and well‐being changes among the parent carer population.
Methods	Eight parent carers were involved, seven of whom had previously participated in the Healthy Parent Carer programme as participants (six) and facilitators (one); one member had not participated in HPC. Group meetings were co‐designed and facilitated between two researchers and the Family Involvement Coordinator (who is also a parent carer) to support input from parent carers relating to the study aims design, including sampling and recruitment strategies. Six group online meetings were convened. With two one‐on‐one meetings arranged separately on two occasions. As data began to be collected and analysed, the preliminary insights and findings were discussed with the group to sense‐check, challenge the assumptions of the researchers, and build a more robust representation of the data. PPI members were also involved outside of the group meetings in reviewing study documents, such as the Participant Information Sheet, study advert and consent form.
Results	PPI contributed to the study in several ways: Setting study priority. Providing advice on sampling and recruitment strategy. Helping to develop the study topic guide through pilot group interviews and feedback to enable us to refine the questions. Contributing to analytical development, where discussion invited parent carers in the PPI group to challenge and build on emerging analysis and coding framework, which included placing greater emphasis upon the effect of the parent carer identity, social isolation, comparison and confidence on health and well‐being outcomes, alongside stress, sleep deprivation and low mood. Dissemination of findings, with the PPI group helping to develop a plain English summary.
Discussion and conclusions	PPI was highly influential in multiple areas as described. In particular, the early analytical framework was clarified surrounding the core challenges parent carers face relating to health and well‐being. Greater emphasis and relevance were placed on the influence of the parent carer identity and confidence, as this resonated strongly with the PPI group through in‐depth discussion.
Reflections/critical perspective	The study data collection was rapid in nature which potentially impacted upon the development of analysis over time with the PPI group. Nevertheless, the PPI group requested a follow‐up meeting to ensure that the full analysis was discussed in sufficient depth.

### 2.2 Participants

Parent carers of children with special educational needs and disabilities (SEND) living in England were eligible. We emailed HPC participants and Lead and Assistant Facilitators who had previously consented to be contacted about research opportunities. We emailed adverts to 200 parent carers in our Family Faculty who had expressed an interest in helping with research. In addition, we advertised on social media via Facebook and X/Twitter, as well as on the HPC website. All adverts included a link to the study website that provided participant information sheets and an online form where potential interviewees could register their interest and provide their contact details and demographic data.

We used maximum‐variation purposive sampling to screen and select interviewees from those who expressed interest in participating. Insofar as it was possible, we sampled using demographic characteristics including sex, ethnicity and residential area deprivation derived from postcodes using the English Indices of Multiple Deprivation 2019 [21], and parents of children with different types of conditions. Informed consent to participate was recorded before each interview. After the interview, interviewees were offered a £35 shopping voucher.

### 2.3 Data Collection

Two researchers (C.Mc.G./P.H.) conducted individual, semi‐structured interviews via a single one‐off video call, approximately 1 h. A topic guide was piloted with our PPI group. The topic guide included two parts; in the first, participants were asked open‐ended questions about health and well‐being, enabling them to discuss topics and experiences without being overly constrained by a pre‐defined structure (Supporting Document 1). In the second part, we conducted an elicitation exercise, in which the interviewees were asked to consider the aspects of health assessed by the 14‐item version of the WEMWBS [14, 15]. Participants were shown the item topics on screen and asked to discuss which—if any—were meaningful to them. Further probing enabled a deeper understanding of their responses, and in the second part, they could refer to the examples shared in the earlier open discussion. Interviewers wrote summaries and reflections following each interview. The interviews were audio‐recorded and transcribed verbatim. The transcripts were uploaded to NVivo qualitative data management software for coding.

### 2.4 Data Analysis

The first phase of our analysis was an inductive thematic analysis [22]. Three researchers independently coded a sub‐sample of the same transcripts (C.Mc.G. and P.H. *n* = 5 each, A.B. *n* = 4), before arranging the codes into categories and agreeing on a codebook. C.Mc.G. and P.H. used this codebook to analyse the remaining transcripts. We then identified data‐driven themes that we have reported [18].

Subsequently, we undertook a further deductive stage of our analysis that constitutes the substantive novel research in this paper. We used a published framework of well‐being domains and dimensions to enable a more granular analysis and transferable interpretation of key outcome dimensions for parent carers [19]. This framework was selected because it is derived from a comprehensive and systematic synthesis of 99 generic measures of well‐being [19]. The framework lists 196 discrete well‐being dimensions grouped into seven themes (which we call domains to distinguish from the output of thematic analysis): ‘mental wellbeing’, ‘social wellbeing’, ‘physical wellbeing’, ‘spiritual wellbeing’, ‘personal circumstances’, ‘activities and functioning’, and ‘wellbeing overall’. Pragmatically, we excluded the ‘global wellbeing’ domain and the ‘wellbeing overall’ dimension as we thought these were too generic and undefined for our analytical purpose.

One researcher (C.Mc.G.) used NVivo to map our data onto the well‐being framework by comparing the data‐driven categories developed in the first stage of our analysis for fit with the six framework‐driven domains, and then with the more specific dimensions making up each domain. We reviewed the categories and data within each domain and dimension collectively to ensure an appropriate and acceptable fit. We utilised the Supporting Appendix the authors of the framework provided, which includes a brief description of each dimension, as they noted potential differences with ‘dictionary definitions or current thinking’ [19]. To arrive at the final list of the salient dimensions, pragmatically, we set the following criteria for assessment:

i. Data could be found that supported the dimension;
ii. Interviewees implicitly or explicitly emphasised the importance of the dimension;
iii. Interviewees identified and discussed the importance of the dimension specifically for parent carers.

We used an iterative process of paring and consolidation based on the above criteria. Paring involved incrementally removing framework dimensions when no supporting data were found. Consolidation involved merging dimensions with significant conceptual overlap, that is, where most data could fit into both dimensions. Two researchers (C.Mc.G. and P.H.) developed a shortlist of the most salient dimensions using this process and the criteria. We discussed the emerging list amongst ourselves and with members of our PPI group. Following final sense‐checking and discussion, we agreed on the more important health and well‐being dimensions for parent carers and considered the appropriateness of WEMWBS as a measure of well‐being for parent carers.

### 2.5 Ethics Approval

The study was approved by the University of Exeter Medical School Research Ethics Committee (UEMS REC ID: 525009).

### 2.6 Results

We interviewed 30 parent carers (Table 2). Fourteen interviewees had participated in the HPC programme, five were HPC Lead or Assistant Facilitators, and eleven had no prior connection to the HPC programme.

**Table 2:** Characteristics of interviewed parent carers (*n* = 30).

		*n*	%
Parent carer mean age—years (range)		47 (33–76)	
Parent carer sex	Female	24	80.0
	Male	6	20.0
Partner at home	Yes	24	80.0
	No	6	20.0
Parent carer ethnicity	Asian, Asian British	4	13.3
	White	25	83.3
	Mixed	1	3.3
Parent education	Degree or above	18	60.0
	Finished education at 18 years old	7	23.3
	Finished education at 16 years old	5	16.6
Previous participation in Healthy Parent Carers (HPC)	Facilitators in HPC	5	16.7
	Participants in HPC	14	46.7
	Non‐participants in HPC	11	36.7
Child's main diagnosis	Autism	24	58.9
	Autism with ADHD	4	9.8
	Learning disability	4	9.8
	Severe congenital neuromotor disorder	4	9.8
	Cerebral palsy	2	4.9
	Down syndrome	1	2.4
	Chronic Fatigue Syndrome	1	2.4
	Undiagnosed (social and communication difficulties)	1	2.4
Child's mean age—years (range) (*n* = 41)		12.8 (3–28)	
Deprivation in the area of residence (Indices of multiple deprivation—IMD; quintiles)	1 (most deprived)	6	20.0
	2	6	20.0
	3	5	16.7
	4	7	23.3
	5 (least deprived)	6	16.7

We found support for diverse dimensions across all six well‐being domains. Our results are organised into the six domains, with a list of the proposed ‘most important’ dimensions to parent carers within these domains presented in Table 3.

**Table 3:** The most important health and well‐being dimensions for parent carers.

Domain	Dimension
Mental well‐being	Stress reaction Anxiety/depression Acceptance Autonomy Self‐esteem Cognition
Activities and functioning	Achievement Interests/hobbies Learning
Social well‐being	Need for relatedness
Physical well‐being	Relaxation Sleep Fitness
Spiritual well‐being	Life purpose and satisfaction
Personal circumstances	Community well‐being Status Financial situation Future security

We illustrate the findings with quotes, attributed with participant numbers and indicating whether they were HPC participants (P), HPC facilitators (F), or had not participated in the HPC programme (NP).

### 2.7 Mental Well‐Being

The mental well‐being domain stood out to parent carers. In particular, the ‘stress reaction’ dimension was highlighted; parent carers felt that they had very little time to fulfil all of their responsibilities.

‘It's the stress of fitting too much in. That's the stress…. We have all these responsibilities, sure, but in the same 24/7 hours that the rest of the world has…. There's just not enough time.’

F‐19

The ‘anxiety’ dimension was pertinent in relation to parental uncertainty around whether their child support needs would be met in the future. The ‘depression’ dimension was significant, especially through connection to the ‘social isolation’ and ‘hope’ dimensions. Furthermore, some parent carers felt hopeless and socially isolated because caring for others dominated their lives, which led them to feel like they were losing their identity. Data within the ‘mental health/symptoms’ dimension highlighted the mental health impact of being a parent carer, with some interviewees describing suicidal ideation.

‘I think a lot of parent carers have had suicidal thoughts…. They've literally felt like they have no life, that their life has no … that they don't really have an existence. It's that bad.’

P‐5

These findings suggest that improvement in these dimensions of mental health would reflect improved health and well‐being in areas that are a key priority for parent carers.

The ‘acceptance’ and ‘autonomy’ dimensions reflected how parent carers understood those concepts from their own experiences, such as gaining confidence to make one's own decisions. These dimensions were also linked because ‘acceptance’ was seen as a means of determining their locus of control and, therefore, what goals could be prioritised. Data which supported the ‘cognition’ dimension suggested that calmness, clarity of thought and problem‐solving are essential valued outcomes for parent carers.

‘And then I deal better with problems. Because I'm calm. I'm calm, and I'm thinking to myself, “It's actually not worth it,” and “you have to take the opportunities that are there, so stay cool.” I can deal better with problems because I am making much more logical decisions because I'm calmer.’

P‐18

Aspects of positive mental well‐being dimensions, such as ‘acceptance’, ‘autonomy’, ‘confidence/self‐esteem’ and ‘cognition’, featured more among parent carers who had taken part in or facilitated the HPC programme. Aspects of growing ‘self‐esteem’ were discussed by interviewees describing how the HPC programme had changed their perspective in these areas. They suggested that both the course content and the social aspect of being in group sessions contributed to this positive shift in perspective. The ‘self‐care’ dimension was a salient dimension for parent carers, who linked this dimension to increased confidence and self‐esteem, as well as to increased autonomy.

‘Maybe it comes back to feeling more confident to do something, but actually it is a little bit more than that. Maybe it comes back to that idea of empowerment to take control and say, “I'm going to look after myself in order to improve things.”’

F‐8

### 2.8 Activities and Functioning

Overall, our data indicated that freeing up resources of time and energy allowed parent carers to experience positive changes in the ‘activities and functioning’ domain. Parent carers who participated in the HPC programme engaged more in activities outside of their caring responsibilities, such as ‘learning’, experiencing ‘achievement’ and pursuing ‘interests/hobbies’. However, some parent carers described limits on their capacity to pursue such activities, which had a negative impact on their well‐being.

‘The issue is that this person is stuck at home with children that have got a huge amount of need and they've got no support, they've got no friends, they can't get out and they are that hamster on a wheel and they're just desperate to get off.’

F‐5

### 2.9 Social Well‐Being

The ‘need for relatedness’ dimension appeared to be hugely significant within the social well‐being domain. Interviewees who had facilitated or participated in the HPC programme reflected how this dimension had changed in a positive way because of taking part in the programme.

‘I think before coming onto the programme … everybody felt quite isolated and they didn't feel like they had any connections outside of their family unit.’

F‐11

Participants who had been involved in the HPC programme talked about positive changes to social well‐being. In addition to the crucial ‘need for relatedness’, their responses also spoke to the ‘praise and respect from others’ and ‘social acceptance’ dimensions. In some contexts, social comparisons and social isolation seemed to mediate social well‐being. When parent carers were not in contact with other parent carers, social comparisons appeared to have a negative impact. However, when parent carers spent time with other parent carers, comparisons had a more positive impact. This impact was augmented by peer support and the positive implicit and explicit feedback that came with joining a peer‐group intervention.

‘Talking and sharing this with others on the course means that others are going through the same thing. And then when tips are shared, or everyone saying, “It happened to me,” you're feeling a bit better about yourself.’

F‐10

### 2.10 Physical Well‐Being

We found connections in our data with the ‘physical’ and ‘mental wellbeing’ domains, suggesting that parent carers associate an increase in physical and mental well‐being with ‘fitness and exercise’ and ‘physical activity’.

‘A lot of the time it's the simple things. They'll now start going for a walk or something like that…. I think it's the small things that they seem to come back in a really positive way.’

F‐19

Data supported the importance of the ‘rest’, ‘relaxation’ and ‘sleep’ dimensions and indicated that parent carers find ‘relaxation’ achievable within their lifestyle and routines, because relaxation can be undertaken in ‘bitesize chunks.’

‘I think sometimes just a short 10/15 minutes, like having a longer soak in the bath. Just taking bitesize chunks of time—all of these things can just make a difference really.’

P‐1

### 2.11 Spiritual Well‐Being

Aspects of the ‘life purpose and satisfaction’ dimension within the ‘spiritual wellbeing’ domain resonated with our data. While parent carers were proud of their contribution to their family, they also described an increase in ‘life purpose’ when they took on responsibilities outside of caring for their immediate family.

‘Stress was alleviated by having a purpose other than caring for my family … the voluntary work that I was doing had a huge impact on my community and it wasn't the same as giving to my son … it's very important for us as human beings to do things for other people.’

P‐2

‘…it's finding meaning in other places … there were good times with him growing up. There are funny times and there are joyful times as well.’

NP‐15

### 2.12 Personal Circumstances

In the case of the ‘personal circumstances’ domain, the ‘community wellbeing’ dimension appeared important for parent carers because it reflected how they were able to find ways to use their experience to benefit others.

‘I had an opportunity to start volunteering for a Community Interest Company, but I didn't have the confidence to do it. The group said, “Go for it, you need an outlet.” So I applied and I was accepted. Now [I'm?] a director!’

P‐2

The ‘status’ dimension appeared salient because parent carers articulated ‘fighting’ for recognition and resources from professionals and professional bodies—which parent carers described as a disempowering experience, creating feelings of ‘low status’. Finally, parent carers highlighted aspects of the ‘financial situation’ and ‘future security’ because of the resource demands associated with having a child with a disability.

‘The financial impact on the family is massive and yet the financial demands are much higher because—with a child with SEND—everything costs more. That has an effect on their abilities to cope.’

F‐19

### 2.13 Reflections on WEMWBS to Measure Well‐Being of Parent Carers

Parent carers in our study generally agreed that the WEMWBS captured important aspects of well‐being. However, their caregiving roles and experiences affected their interpretation of some concepts and the suitability of WEMWBS items for evaluating parent carer‐focused interventions. For instance, the items ‘feeling useful’ or ‘feeling relaxed’ were seen as contentious and did not fully resonate in their lived experience.

‘I might not have the capacity to be more useful. I might already be doing everything I can.’

P‐4

‘My life is very stressful with my daughter, it's definitely not relaxed when she's around.’

P‐2

Parent carers expressed scepticism about whether ‘feeling loved’ would change significantly because of an intervention, as it depends on changes in the behaviour of individuals other than the parent carers themselves.

‘Maybe feeling more loved [is not appropriate] because that's relying on someone else to be able to do that.’

P‐15

These examples highlight some misalignment between parent carers' lived experience and perception of their health and well‐being compared to WEMWBS content.

Parents were also asked specifically to consider whether they felt anything was missing from the WEMWBS outcome measure. While some parents found it difficult ‘pinpoint’ missing items, other parents considered areas of their lifestyle and context that were not captured in the instrument. Some missing concepts that were named appeared to overlap with other items in the WEMWBS—such as ‘increased motivation’, which appears to directly relate to ‘I've been interested in new things’. On the other hand, ‘giving to others’ was deemed to be missing with sufficient differentiation to ‘I've been feeling useful’. One parent highlighted that ‘*physical health…. I think being active is a really big one for us’* (F‐11), but was not present in the measure.

Rather than identifying a specific domain as missing, some parent carers identified the importance of behaviour change around health‐related activities. The concept of autonomy is integral to the WEMWBS; nevertheless, parent carers in our study felt this was not sufficiently captured within the existing items. For them, autonomy was associated with the process of rediscovering a sense of identity beyond their caregiving role—an insight that underscores the importance of positive self‐identity as a meaningful indicator of well‐being.

‘I think some way of capturing that is really important because that is about self‐identity and how you then be yourself as someone that's able and doing something for you.’

P‐9

The emotional experience of many parent carers, particularly the process of acceptance that is in the well‐being framework, is not reflected in WEMWBS items.

## 3 Discussion

Our findings elucidate the importance of key health and well‐being dimensions to parent carers across all six domains of a comprehensive well‐being framework. In the mental well‐being domain, ‘anxiety/depression’ and ‘stress’ appeared particularly important. In the activities and functioning domain, ‘achievement’, ‘interests and hobbies’ and ‘learning’ were prominent. Within the social well‐being domain, ‘need for relatedness’ and ‘social isolation’ were important. In the physical well‐being domain, ‘rest’, ‘sleep’ and ‘physical exercise’ were significant. In spiritual well‐being, ‘life purpose and satisfaction’ were salient. In personal circumstances, ‘status’ and ‘financial situation’ dimensions featured prominently.

By design, WEMWBS items cover both hedonic and eudaimonic aspects of mental health, including positive affect, that is, the positive aspects of mental health, to avoid ceiling effects [14]. Our findings indicate that parent carers are unlikely to reach these ‘ceilings’ due to the persistent challenges they face. Therefore, not measuring reductions in ‘negative’ well‐being dimensions risks failing to detect some of the most important improvements in health and well‐being. Furthermore, some parent carers felt that certain concepts within the WEMWBS did not fully resonate with their lived experience and well‐being. Mental well‐being concerns like anxiety, depression and stress were significant and closely connected with social well‐being, including relatedness and isolation, while physical well‐being, such as sleep, rest and exercise, were also important. It is our contention that current well‐being measures, such as WEMWBS, may not fully capture parent carers' lived experiences.

Our study addresses the uncertainty between the subjective experiences of parent carers and the measures traditionally used to assess their well‐being. Previous research has identified that parent carers face a higher risk of poorer health, social isolation and financial strain, but few studies have directly explored their lived experiences to refine appropriate outcome measures. Our work strengthens the argument for PROMs that reflect what truly matters to parent carers, ensuring that interventions designed to support them are evaluated using dimensions that accurately capture the state of, and changes in, their well‐being [23, 24, 25].

Several of the important dimensions highlighted by parent carers in our data reflect symptoms of ill health. For example, stress, anxiety and depression are widely implicated as negative mental health outcomes for parent carers. According to proponents of the ‘stress‐health’ model, stress has been found to significantly impact carers' health and well‐being due to stressors common to this group, such as the assessment and diagnosis journey [26]. Of concern is suicidal ideation amongst parent carers, which previous research connected with parent carer anxiety and depression, and in some cases can lead to disproportionate numbers of deaths by suicide in carers [27].

Within the ‘personal circumstances’ domain, we found that the ‘financial situation’ and ‘future security’ dimensions were especially relevant because of the financial burdens parent carers often experience [28]. Data which supported the ‘sleep’ and ‘relaxation’ dimensions showed that parent carers' physical well‐being needs often go unmet, aligning with previous research that emphasises the importance of sleep for parent carers [29]. Other research has highlighted that adverse physical health outcomes, such as headaches and musculoskeletal pain, may be associated with caring responsibilities [30].

Furthermore, our findings underscore the need for interventions, such as the HPC and other parent carer‐focused programmes, to promote the health and well‐being of parent carers. Parent carers who engaged in these interventions reported improvements in self‐esteem, autonomy, social connection and engagement in activities outside of their caring responsibilities—factors that contribute to long‐term resilience. These insights reinforce the importance of designing support systems that not only address immediate health concerns but also empower parent carers to cultivate sustainable well‐being.

Our study highlights dimensions of health and well‐being that can drive positive behaviour change through targeted interventions, making them essential for future measurement. For example, ‘praise and respect from others’ and ‘social acceptance’ were especially valued by parent carers. Some parent carers experience limited opportunities for autonomous decision‐making, a factor associated with poorer well‐being outcomes in the literature [31]. Participation in programmes that foster greater autonomy, encouraging parent carers to engage in learning, hobbies and work‐related achievements, is likely to enhance well‐being but requires salient outcome measures to capture meaningful change.

In this paper, we built on our previous inductive analysis by using a deductive analytic approach to ensure that the findings were grounded in the data and interpreted using a comprehensive well‐being framework. The framework that we used provided a systematically derived ‘inventory of wellbeing dimensions’, derived from an interdisciplinary understanding of well‐being. This framework enabled us to elucidate the dimensions of health and well‐being that are most important to parent carers. Refinement through pragmatic consensus among our team and triangulation in consultation with parent carers in our PPI group helped ensure rigour. Our findings were broadly consistent across HPC facilitators, HPC participants and HPC non‐participants and also supported by wider literature. However, we found that some aspects of positive mental well‐being seemed more prominent amongst HCP facilitators and participants, which might have reflected their experiences specific to the programme.

Despite efforts to recruit a diverse sample, more participants were parents of children with autism than with physical disabilities. Nearly 80% of participants were white British, limiting transferability to other ethnic populations. However, British Asian parent carers in the PPI group noted that key concerns were similar across ethnicities, though cultural differences, such as the emphasis on spiritual well‐being, may influence well‐being priorities.

By analysing participants' perspectives systematically through the framework‐driven approach, we identified key well‐being dimensions that should be prioritised when measuring health and well‐being outcomes in this population. Nevertheless, the paring and consolidation process we used to link our data to the dimensions was undertaken systematically but also required pragmatic decisions. There were instances where interpretations of narrative quotes could have been categorised across different or multiple dimensions. The final decision on the most important outcomes was taken by us as the researchers. Alternative approaches that could build on this could involve engaging stakeholders in ranking.

By integrating qualitative methods with a structured well‐being framework, this study provides a robust foundation for future research seeking to evaluate and/or improve the health and well‐being of parent carers. There is a growing number of parent‐carer‐focused interventions and associated evaluative research. The WEMWBS is the primary outcome measure for one large, randomised controlled trial currently in progress, though each includes secondary outcome measures assessing anxiety and depression [32], whereas another large trial uses the General Health Questionnaire to quantify caregiver mental health employing WEMWBS as a secondary outcome measure [33].

Further implications for research recommendations include that identifying the most salient aspects of health and well‐being to parent carers as a group potentially lays the foundations for the development of a parent carer population‐specific PROMs. Similarly, there is scope for these findings to inform development and consensus on a core outcome set [34]. Identifying the important health and well‐being dimensions as targets can guide policy to better support and shape the design of targeted interventions that improve health‐related quality of life. Ultimately, ensuring that research and policy align with people's lived experiences is essential for fostering a more inclusive and responsive approach.

Our research provides a worked methodological example of conducting the sort of due diligence that may be necessary for researchers to ensure the PROMs selected for measuring outcomes in evaluative studies meet the criterion of appropriateness adequately. This is vital for the primary outcome measure in trials on which sample sizes are calculated, and the magnitude of change in scores determines whether an intervention is effective.

## 4 Conclusion

Our study highlights specific health and well‐being dimensions that are meaningful to parent carers across six domains. Focusing on the specific health and well‐being dimensions identified as meaningful to parent carers in this paper is a helpful step to future co‐design of appropriate PROM development for this population.

## Author Contributions

**Caomhán McGlinchey:** data curation, formal analysis, investigation, methodology, project administration, validation, writing – original draft, writing – review and editing, visualisation. **Phillip Harniess:** data curation, formal analysis, investigation, methodology, project administration, validation, writing – original draft, writing – review and editing, visualisation. **Aleksandra J. Borek:** conceptualisation, data curation, formal analysis, funding acquisition, investigation, methodology, project administration, supervision, validation, writing – original draft, writing – review and editing. **Alice Garrood:** project administration, writing – review and editing, methodology. **Annabel McDonald:** funding acquisition, project administration, formal analysis, writing – review and editing. **Fleur Boyle:** project administration, writing – review and editing. **Stuart Logan:** conceptualisation, supervision, writing – review and editing. **Christopher Morris:** conceptualisation, data curation, formal analysis, funding acquisition, investigation, methodology, project administration, supervision, validation, writing – original draft, writing – review and editing.

## Disclosure

The authors have nothing to report.

## Ethics Statement

The study was approved by the University of Exeter Medical School Research Ethics Committee (UEMS REC ID: 525009).

## Conflicts of Interest

The authors declare no conflicts of interest.

## Supporting information

R1‐HE‐Supplementary document 1_Topic guide.

## Data Availability

The data that support the findings of this study are available from the corresponding author upon reasonable request.

## References

[1] J. C. Brehaut , R. E. Garner , A. R. Miller , et al., “Changes Over Time in the Health of Caregivers of Children With Health Problems: Growth‐Curve Findings From a 10‐Year Canadian Population‐Based Study,” American Journal of Public Health 101, no. 12 (2011): 2308–2316. 22021302 10.2105/AJPH.2011.300298 PMC3222435

[2] L. K. Fraser , F. E. Murtagh , J. Aldridge , T. Sheldon , S. Gilbody , and C. Hewitt , “Health of Mothers of Children With a Life‐Limiting Condition: A Comparative Cohort Study,” Archives of Disease in Childhood 106, no. 10 (2021): 987–993. 33653713 10.1136/archdischild-2020-320655 PMC8461446

[3] S. C. Masefield , S. L. Prady , T. A. Sheldon , N. Small , S. Jarvis , and K. E. Pickett , “The Caregiver Health Effects of Caring for Young Children With Developmental Disabilities: A Meta‐Analysis,” Maternal and Child Health Journal 24 (2020): 561–574. 32048172 10.1007/s10995-020-02896-5 PMC7170980

[4] E. Coulman , N. Gore , G. Moody , et al., “Early Positive Approaches to Support (E‐PatS) for Families of Young Children With Intellectual Disability: A Feasibility Randomised Controlled Trial,” Frontiers in Psychiatry 12 (2021): 729129. 34992552 10.3389/fpsyt.2021.729129 PMC8725992

[5] F. Martin , W. Clyne , G. Pearce , and A. Turner , “Self‐Management Support Intervention for Parents of Children With Developmental Disorders: The Role of Gratitude and Hope,” Journal of Child and Family Studies 28 (2019): 980–992.

[6] H. M. Bourke‐Taylor , F. Jane , and J. Peat , “Healthy Mothers Healthy Families Workshop Intervention: A Preliminary Investigation of Healthy Lifestyle Changes for Mothers of a Child With a Disability,” Journal of Autism and Developmental Disorders 49 (2019): 935–949. 30377884 10.1007/s10803-018-3789-1

[7] A. J. Borek , B. McDonald , M. Fredlund , G. Bjornstad , S. Logan , and C. Morris , “Healthy Parent Carers Programme: Development and Feasibility of a Novel Group‐Based Health‐Promotion Intervention,” BMC Public Health 18 (2018): 270. 29458355 10.1186/s12889-018-5168-4 PMC5819077

[8] A. Garrood , G. Bjornstad , A. Borek , et al., “Healthy Parent Carers: Acceptability and Practicability of Online Delivery and Learning Through Implementation by Delivery Partner Organisations,” Health Expectations 26, no. 5 (2023): 2050–2063. 37401625 10.1111/hex.13812 PMC10485339

[9] J. Reeder , M. H. P. Rogers , F. Shamsaddin , et al., Interventions Supporting the Empowerment of Parent Carers of Children With Long‐Term Health Conditions: A Scoping Review (2025). 10.1111/dmcn.70039 PMC12982629 41139287

[10] L. B. Mokkink , C. B. Terwee , D. L. Patrick , et al., “The COSMIN Study Reached International Consensus on Taxonomy, Terminology, and Definitions of Measurement Properties for Health‐Related Patient‐Reported Outcomes,” Journal of Clinical Epidemiology 63, no. 7 (2010): 737–745, 10.1016/j.jclinepi.2010.02.006. 20494804

[11] I. Chalmers and P. Glasziou , “Avoidable Waste in the Production and Reporting of Research Evidence,” Lancet 374, no. 9683 (2009): 86–89. 19525005 10.1016/S0140-6736(09)60329-9

[12] R. Fitzpatrick , C. Davey , M. J. Buxton , et al., “Evaluating Patient‐Based Outcome Measures for Use in Clinical Trials” *Health Technol Assess* 2, no.14 (1998). 9812244

[13] G. Bjornstad , K. Wilkinson , B. Cuffe‐Fuller , et al., “Healthy Parent Carers Peer‐Led Group‐Based Health Promotion Intervention for Parent Carers of Disabled Children: Protocol for a Feasibility Study Using a Parallel Group Randomised Controlled Trial Design,” Pilot and Feasibility Studies 5 (2019): 137. 31788323 10.1186/s40814-019-0517-3 PMC6875041

[14] R. Tennant , L. Hiller , R. Fishwick , et al., “The Warwick‐Edinburgh Mental Well‐Being Scale (WEMWBS): Development and UK Validation,” Health and Quality of Life Outcomes 5 (2007): 63. 18042300 10.1186/1477-7525-5-63 PMC2222612

[15] W. Deng , S. Carpentier , J. Blackwood , and A. Van de Winckel , “Rasch Validation of the Warwick‐Edinburgh Mental Well‐Being Scale (WEMWBS) in Community‐Dwelling Adults,” BMC Psychology 11, no. 1 (2023): 48. 36803574 10.1186/s40359-023-01058-w PMC9936469

[16] G. Bjornstad , B. Cuffe‐Fuller , O. C. Ukoumunne , et al., “Healthy Parent Carers: Feasibility Randomised Controlled Trial of a Peer‐Led Group‐Based Health Promotion Intervention for Parent Carers of Disabled Children,” Pilot and Feasibility Studies 7 (2021): 144. 34301334 10.1186/s40814-021-00881-5 PMC8298691

[17] J. Lloyd , G. Bjornstad , A. Borek , et al., “Healthy Parent Carers Programme: Mixed Methods Process Evaluation and Refinement of a Health Promotion Intervention,” BMJ Open 11, no. 8 (2021): e045570. 10.1136/bmjopen-2020-045570 PMC8388296 34433591

[18] C. McGlinchey , P. Harniess , A. J. Borek , et al., “What Aspects of Health and Wellbeing Are Most Important to Parent Carers of Children With Disabilities?,” Health Expectations 27, no. 3 (2024): e14085. 38845158 10.1111/hex.14085 PMC11156688

[19] M. J. Linton , P. Dieppe , and A. Medina‐Lara , “Review of 99 Self‐Report Measures for Assessing Well‐Being in Adults: Exploring Dimensions of Well‐Being and Developments Over Time,” BMJ Open 6, no. 7 (2016): e010641, 10.1136/bmjopen-2015-010641. PMC4947747 27388349

[20] S. Staniszewska , J. Brett , I. Simera , et al., “GRIPP2 Reporting Checklists: Tools to Improve Reporting of Patient and Public Involvement in Research,” BMJ 358 (2017): j3453, 10.1136/bmj.j3453. 28768629 PMC5539518

[21] S. Noble , D. McLennan , M. Noble , et al., *The English Indices of Deprivation 2019* (Ministry of Housing, Communities and Local Government, 2019).

[22] V. Braun and V. Clarke . Thematic Analysis: A Practical Guide. 2021.

[23] C. M. Potter , L. Batchelder , C. A'Court , et al., “Long‐Term Conditions Questionnaire (LTCQ): Initial Validation Survey Among Primary Care Patients and Social Care Recipients in England,” BMJ Open 7, no. 11 (2017): e019235, 10.1136/bmjopen-2017-019235. PMC5695378 29101153

[24] C. M. Potter , M. Peters , M. Cundell , R. McShane , and R. Fitzpatrick , “Use of the Long‐Term Conditions Questionnaire (LTCQ) for Monitoring Health‐Related Quality of Life In People Affected by Cognitive Impairment Including Dementia: Pilot Study in UK Memory Clinic Services,” Quality of Life Research 30, no. 6 (2021): 1641–1652, 10.1007/s11136-021-02762-z. 33575918 PMC8178132

[25] C. M. Potter , M. Peters , M. Cundell , R. McShane , and R. Fitzpatrick , “Living Well While Providing Support: Validation of LTCQ‐Carer for Assessing Informal Carers' Quality of Life,” Quality of Life Research 32, no. 12 (2023): 3507–3520, 10.1007/s11136-023-03485-z. 37530960 PMC10624753

[26] M. E. Warfield , M. W. Krauss , P. Hauser‐Cram , C. C. Upshur , and J. P. Shonkoff , “Adaptation During Early Childhood Among Mothers of Children With Disabilities,” Journal of Developmental and Behavioral Pediatrics: JDBP 20, no. 1 (1999): 9–16. 10071940 10.1097/00004703-199902000-00002

[27] S. T. O'Dwyer , A. Janssens , A. Sansom , et al., “Suicidality in Family Caregivers of People With Long‐Term Illnesses and Disabilities: A Scoping Review,” Comprehensive Psychiatry 110 (2021): 152261. 34332205 10.1016/j.comppsych.2021.152261

[28] C. M. Blackburn , N. J. Spencer , and J. M. Read , “Prevalence of Childhood Disability and the Characteristics and Circumstances of Disabled Children in the UK: Secondary Analysis of the Family Resources Survey,” BMC Pediatrics 10 (2010): 21. 20398346 10.1186/1471-2431-10-21 PMC2873517

[29] J. Lee , “Maternal Stress, Well‐Being, and Impaired Sleep in Mothers of Children With Developmental Disabilities: A Literature Review,” Research in Developmental Disabilities 34, no. 11 (2013): 4255–4273. 24080069 10.1016/j.ridd.2013.09.008

[30] J. Fairthorne , N. de Klerk , and H. Leonard , “Health of Mothers of Children With Intellectual Disability or Autism Spectrum Disorder: A Review of the Literature,” Medical Research Archives 1, no. 3 (2015).

[31] J. Van Assche , J. van der Kaap‐Deeder , E. Audenaert , M. De Schryver , and M. Vansteenkiste , “Are the Benefits of Autonomy Satisfaction and the Costs of Autonomy Frustration Dependent on Individuals' Autonomy Strength?,” Journal of Personality 86, no. 6 (2018): 1017–1036, https://onlinelibrary.wiley.com/doi/10.1111/jopy.12372. 29377144 10.1111/jopy.12372

[32] N. Gore . Early Positive Approaches to Support (E‐PAtS) for Families of Young Children With Intellectual Disability: A Randomised Controlled Trial. International Standard Randomised Controlled Trial Number Registry: BioMed Central Ltd, Springer Nature, 2025, https://www.isrctn.com/ISRCTN11192967.

[33] K. Leadbitter , R. Smallman , K. James , et al., “REACH‐ASD: A UK Randomised Controlled Trial of a New Post‐Diagnostic Psycho‐Education and Acceptance and Commitment Therapy Programme Against Treatment‐as‐Usual for Improving the Mental Health and Adjustment of Caregivers of Children Recently Diagnosed With Autism Spectrum Disorder,” Trials 23, no. 1 (2022): 585, 10.1186/s13063-022-06524-1. 35869533 PMC9306249

[34] P. R. Williamson , D. G. Altman , J. M. Blazeby , et al., “Developing Core Outcome Sets for Clinical Trials: Issues to Consider,” Trials 13 (2012): 132. 22867278 10.1186/1745-6215-13-132 PMC3472231

