# Supplementary material for: What Are the Important Health and Well‐Being Outcome Dimensions for Parent Carers of Disabled Children? A Qualitative Study
Source: Health Expect. 2025 Jul 29;28(4):e70358. doi: 10.1111/hex.70358 (PMC12304521; doi:10.1111/hex.70358)
Supplement: Supplementary file 1 — R1‐HE‐Supplementary document 1_Topic guide. [file HEX-28-e70358-s001.docx]

# **“What are the important health and wellbeing outcome dimensions for parent carers of disabled children? A qualitative study”**

# **Supplementary Document 1 – Interview Topic Guide**

**Housekeeping and introductions**

- Welcome and thank the participant for agreeing to take part.
- Researcher introduce oneself.
- Recap the aim of the study.
- Check that participants are still happy to consent (e-consent will be obtained before the start of the interview) and if they have any questions.
- Recap the right to pause or stop the interview.
- Ask for permission to start the audio-recorder.

**List of topics with example questions**

1. **Experiences of parent carer programmes, e.g.:**
   1. *Have you had experience with any structured parent carer focused programmes?* (Prompt with examples if necessary.)
   2. *What was your experience? To what extent did you perceive any benefits of the programme? (*Prompt if they can describe any positive or negative outcomes for them and their health.)
   3. *Which aspects of health and wellbeing do you think are difficult for parent carers?*
   4. *Which aspects of physical and mental health should be targets for parent carer focused programmes?* (Probe further about physical and separately about mental health.)
2. **Reflections on the topics asked about in health and wellbeing questionnaire (i.e. Warwick-Edinburgh Mental Wellbeing Scale), e.g.:**

*Please read and reflect on the 14 topics asked about in a standardised questionnaire that is a way to measure mental health and wellbeing*

*[Show slides of Warwick Edinburgh Mental wellbeing scale items].*

1. *Do these topics resonate for you as a parent carer?*
2. *Do the questions reflect ways in which you felt your health and wellbeing was improved by participating in Healthy Parent Carers [or another similar programme]?*
3. *Do the questions reflect ways in which you felt your health and wellbeing was improved other structured parent carer focused programmes?*
4. *Which topics are the most relevant or meaningful to you and why?*
5. *Which topics appear the least relevant or meaningful to you and why?*
6. *Are there any topics related to your health and wellbeing that are missing?*
7. **Summary**
   1. *Which aspects of health do you think are most important to assess the health and wellbeing of parent carers?*
   2. *Overall, do you have any other thoughts or suggestions on how to assess the health and wellbeing of parent carers participating in structured health programmes?*
   3. Is there anything else you’d like to say that we haven’t covered?
